# SEOM-GECP-GETTHI Clinical Guidelines for the treatment of patients with thymic epithelial tumours (2021)

**DOI:** 10.1007/s12094-022-02788-w

**Published:** 2022-02-05

**Authors:** J. Remon, R. Bernabé, P. Diz, E. Felip, J. L. González-Larriba, M. Lázaro, X. Mielgo-Rubio, A. Sánchez, I. Sullivan, B. Massutti

**Affiliations:** 1 Department of Medical Oncology, Centro Integral Oncológico Clara Campal (HM-CIOCC), Hospital HM Nou Delfos, HM Hospitales, Avinguda de Vallcarca, 151, 08023 Barcelona, Spain; 2 Department of Medical Oncology, Hospital Universitario Virgen del Rocío, Seville, Spain; 3 Department of Medical Oncology, Hospital Universitario de León, León, Spain; 4 Department of Medical Oncology, Vall d’Hebron University Hospital and Vall d’Hebron Institute of Oncology (VHIO), Barcelona, Spain; 5 Department of Medical Oncology, Hospital Universitario Clínico San Carlos, Madrid, Spain; 6 Department of Medical Oncology, Hospital Alvaro Cunqueiro, Vigo, Spain; 7 Department of Medical Oncology, Hospital Universitario Fundación Alcorcón, Madrid, Spain; 8 Department of Medical Oncology, Consorcio Hospitalario Provincial de Castellón, Castellón de la Plana, Spain; 9 Department of Medical Oncology, Hospital de la Santa Creu i Sant Pau, Barcelona, Spain; 10 Department of Medical Oncology, Hospital General Universitario de Alicante, Alicante, Spain

**Keywords:** Thymic epithelial tumours, Chemotherapy, Lenvatinib, Nivolumab, Multidisciplinary

## Abstract

Thymic epithelial tumours (TET) represent a heterogeneous group of rare malignancies that include thymomas and thymic carcinoma. Treatment of TET is based on the resectability of the tumour. If this is considered achievable upfront, surgical resection is the cornerstone of treatment. Platinum-based chemotherapy is the standard regimen for advanced TET. Due to the rarity of this disease, treatment decisions should be discussed in specific multidisciplinary tumour boards, and there are few prospective clinical studies with new strategies. However, several pathways involved in TET have been explored as potential targets for new therapies in previously treated patients, such as multi-tyrosine kinase inhibitors with antiangiogenic properties and immune checkpoint inhibitors (ICI). One third of patient with thymoma present an autoimmune disorders, increasing the risk of immune-related adverse events and autoimmune flares under ICIs. In these guidelines, we summarize the current evidence for the therapeutic approach in patients with TET and define levels of evidence for these decisions.

## Methodology

These guidelines are based on leading studies published in peer review journals. The Infectious Diseases Society of America grading system was used to assign levels of evidence and grades of recommendation [1].

## Epidemiology

Thymic epithelial tumours (TET) represent a group of rare, heterogeneous malignancies arising from thymic epithelial cells, and represent 50% of all anterior mediastinal masses. TET entities include thymomas (T) with subtypes (A, AB, B1, B2, B3) and aggressive thymic carcinomas (TC) [2]. In the European Union, the overall annual incidence of TET is 0.18 per 100.000 (T: 0.14/100.000, and TC: 0.01/100.000) [3]. The overall incidence in Spain remains unknown, but as an example, in Madrid 18 new cases were reported in 2019 [4]. The incidence of TETs is slightly higher in men than women (1.4:1), and increases with age, reaching a peak in the seventh decade of life. However, among Afro-Americans the incidence is higher in younger people than among whites [5]. There are no identified risk factors for developing TETs. However, a higher incidence of TETs has been reported in multiple endocrine neoplasia type 1 [6]. Similarly, in patients with TET, several studies have documented rates of second malignancies ranging from 8 to 31%, particularly thymomas [7]. The prognosis of TET correlates with the histological subtype, with a 5-year overall survival (OS) of ~ 80% and ~ 45% for T and TC, respectively [8]. Due to the rarity of this disease, there are few prospective clinical studies, and most recommendations stem from retrospective cohort studies or expert opinions. (Table 1).

**Table 1:** Recommendations for diagnosis, treatment and follow-up of patients with thymic epithelial tumours

Pathology	–Thymic epithelial tumours are classified according to the WHO histopathological classification –Immunohistochemistry is useful for confirming the diagnosis of TC (CD5 /CD117 positive) [V,A]
Radiology	–The diagnosis of any thymic epithelial tumour relies on a differential diagnosis with other anterior mediastinal tumours and non-malignant thymic lesions –Standard is contrast-enhanced CT scan of the thorax [IV, A] –MRI is recommended in patients with hyperplasia or cystic lesion [IV, B] –PET scan is not generally recommended to assess thymic masses [IV, C]
Baseline biopsy	–Not required if there is high suspicion of thymic epithelial tumour and upfront surgical resection is achievable [IV, E]. Biopsy is required in all other clinical situations [IV, A]
Staging	Post-surgical TET should be routinely staged according to the Masaoka-Koga staging system [III, A] and the 8th edition of the TNM classification [V, A]
Surgery	–Treatment is based on the resectability of the tumour. Surgical resection is the mainstay of treatment if complete resection is deemed to be achievable upfront [IV, A] –Median sternotomy is the standard [IV, A] –Complete thymectomy including the tumour, the residual thymus gland and perithymic fat is preferred [IV, B] –Routine removal of anterior mediastinal and anterior cervical nodes is recommended [IV, A] –Minimally invasive surgery is an option for presumed stage I–II tumours [IV, C] –Surgery of recurrent lesions is recommended if feasible
Radiotherapy	–Postoperative radiotherapy is recommended in stage III, thymic carcinoma and ≥ R1 resection [IV, B] –Post-operative radiotherapy should start within 3 months of complete resection [V, B] –Definitive radiotherapy is recommended as part of a sequential chemoradiotherapy strategy for patients not suitable for surgery or if complete resection is not feasible [III, A]
Perioperative chemotherapy	–Adjuvant chemotherapy is not indicated in thymoma [III, E] and could be considered in thymic carcinoma from stage II –Induction chemotherapy (2–4 cycles) is standard in locally advanced TET [III, A] and PAC is the most common regimen [III, A]. Following that, surgery should be performed if complete resection is deemed achievable
Metastatic disease	–Platinum-based chemotherapy is the standard of care in patients with metastatic disease not suitable for local treatment [III, A] –Complete resection or radical radiotherapy of recurrent lesions is recommended when achievable –There is no standard second line, but carboplatin-paclitaxel, gemcitabine-capecitabine, pemetrexed, or oral etoposide are recommended –Lenvatinib, sunitinib and everolimus [IIIA] are potential targeted therapies –Immunotherapy is not a standard of care
Follow-up	–Baseline CT scan within 3–4 months of surgical resection. [V, C] –For completely resected stage I–II thymoma: annual CT scan for 5 years, then every 2 years. [V, C] –For stage III–IV thymoma, thymic carcinoma or after R1 or R2 resection: CT scan every 6 months for 3 to 5 years, thereafter annually. [V, C] –Continue follow-up for 10–15 years. [V, C]

## Pathological classification

The thymus is composed of lymphocytes and epithelial cells, however,

only the epithelial cells can develop cancer. According to the World Health Organization pathological classification, TET entities include [2]:

- Thymoma A: epithelial cells (at least focally); paucity or absence of immature T cells throughout the tumour.
- Thymoma AB: spindle shaped epithelial cells (at least focally);
- abundance of immature T cells focally or throughout the tumour.
- Thymoma B1: abundance of immature T cells, areas of medullary differentiation; paucity of polygonal or dendritic epithelia cells without clustering.
- Thymoma B2: increased numbers of single or clustered polygonal or dendritic epithelial cells intermingled with abundant immature T cells.
- Thymoma B3: sheets of polygonal slightly to moderately atypical epithelial cells; absent or rare intercellular bridges; paucity or absence of intermingled immature T cells.
- Rare thymoma: thymoma micronodular, metaplastic thymoma.
- Thymic carcinoma: squamous (the most common), basaloid, adenocarcinoma, lymphoepithelioma-like carcinoma and others.
- Neuroendocrine tumours may occur in the thymus, and will not be discussed in these guidelines.

Immunohistochemistry may help in the diagnosis, as cytokeratin 20 is negative in TET, whereas PAX8 is positive, and in contrast to squamous non-small cell lung cancer up to 80% of TCs express either CD5 or CD117. The expression of CD20 occurs in ~ 50% of A or AB thymomas, and GLUT1 expression occurs in ~ 50% of B3 thymoma and TC. Finally, in patients with undifferentiated mediastinal carcinoma, the expression of NUT or inactivation of SMARCA4 could support the diagnosis of middle NUT carcinoma or sarcoma of mediastinum, respectively.

## Diagnosis

### Radiological diagnosis

Standard imaging for thymic tumours is intravenous (i.v.) contrast-enhanced computed tomography (CT) scan of the thorax and abdomen, allowing a complete exploration of the mediastinum and the pleura [IV, A] as a common site of metastatic disease, particularly for thymoma. CT scan is equal or superior to magnetic resonance imaging (MRI), except in cystic lesions [9] [IV, B] and other benign lesions such as thymic hyperplasia. In these cases, MRI is recommended due to the high sensitivity of T1-weighted gradient echo images for detecting microscopic fat [10]. Similarly, MRI could be useful in assessing mediastinal invasion in locally advanced disease with potential surgical options.

18-Fluorodeoxyglucose positron emission tomography (PET)-scan has a sensitivity of 83% and a specificity of 58% [10] for thymus masses, and is not generally recommended [IV, C]. The PET scan can be considered in the cases of TET with aggressive histology, TET in advanced stages to complete the staging work-up or further characterize lesions suspicious for recurrences.

### Baseline pathological assessment

The need for pre-treatment biopsy depends on the resectability of the tumour, and it is not required if the diagnosis of thymic tumour is highly probable and upfront surgical resection is achievable [IV, E]. In other scenarios, either surgical or percutaneous core needle biopsy, which has a sensitivity of up to 90%, is required [11]. Pleural spaces should be respected to avoid tumour cell seeding. Fine-needle aspiration is generally not recommended [IV, D]. [11].

### Autoimmune disorders in thymic epithelial tumours

The thymus gland plays a key role in the development of immune tolerance. Autoimmune disorders (AID) are found in up to 30% of patients with T and in around 3% of patients with TC [12, 13]. In normal conditions, in the thymus those T cells that react against self-antigens are destroyed. However, the AIDs are associated with inactivation of the autoimmune regulator (AIRE) gene within the thymic medulla, hampering the expression of tissue-specific self-antigens that are not recognized by the T cells. This results in multi-organ autoimmune disease due to self-reactive T cells escaping from the thymus and entering the periphery where they can cause autoimmunity [14]. Therefore, as AIDs are not paraneoplastic syndromes, they do not evolve in parallel with tumour evolution.

AID are usually associated with favourable features (i.e., earlier stage of the disease and complete resection status), but they are not an independent prognostic factor for patients with TETs [13]. The most common AID is myasthenia gravis (MG). Up to 30% of patients with T either present with or are eventually diagnosed with MG, whereas, only 10% to 20% of patients with MG present T [13]. MG in TET is seropositive for the acetylcholine receptor antibody, and as these antibodies remain positive irrespective of TET evolution they do not need to be monitored. In some rare cases, TET-related MG is associated with anti-MUSK antibodies. Other AIDs have also been reported in TET, such as Good’s syndrome, pure red cell aplasia, thyroiditis and lupus [12] (Table 2). Therefore, a multidisciplinary approach involving specialists in internal medicine, neurology and others is advised, especially in patients with MG scheduled for surgery.

**Table 2:** Selected autoimmune disorders according to functional organ systems

Neuromuscular	Endocrine
–Myasthenia Gravis	–Thyroiditis
–Peripheral neuropathy	–Autoimmune pituitary diseases
–Encephalomyelitis and limbic encephalitis	–Cushing’s syndrome
–Neuromyotonia (Isaacs’ syndrome)	–Addison’s disease
–Stiff Person syndrome	–Type I diabetes
–Polymyositis	
Haematological	Dermatological
–Pure red cell aplasia	–Pemphigus
–Good’s syndrome	–Lichen planus
–Haemolytic anaemia	–Alopecia areata
–Pernicious anaemia	–Vitiligo vulgaris
–Pancytopenia	
Immune system	Miscellaneous
–Systemic lupus erythematosus	–Glomerulopathies
–Rheumatoid arthritis	–Ulcerative colitis
–Sjogren’s syndrome	–Giant cell myocarditis
–Dermatomyositis/myositis	

## Initial evaluation

The initial evaluation should include radiological studies, a complete history, a full clinical examination (paying particular attention to neurological signs), routine immunological tests, a complete blood cell count with reticulocytes and serum protein electrophoresis, anti-acetylcholine receptor (if positive, electromyogram is not required), and anti-nuclear antibodies tests [V, A]. If other causes of the mediastinal mass are suspected, it is recommended test serum levels of β-human chorionic gonadotropin to rule out seminomas, along with elevated alpha-fetoprotein in non-seminomatous germ-cell tumours. Lymphoma may be considered in patients with rapid onset of B-signs, coexistent lymphadenopathy, or elevated lactate dehydrogenase.

## Staging of thymic epithelial tumours

The most common classification is the Masaoka-Koga staging system [III, A], which correlates with OS. Masaoka-Koga staging can only be performed after surgical resection of the tumour. This classification has been recently updated in a consensus document [15] (Table 3).

**Table 3:** Staging of thymic epithelial tumours: Masaoka-Koga-based staging system [15]

Masaoka-Koga stage	Definition
I	–Grossly and microscopically completely encapsulated tumour including: *Invasion into but not through the capsule *In the absence of capsule, absence of invasion into surrounding tissues
IIA	Microscopic transcapsular invasion (< 3 mm)
IIB	–Gross extension into normal thymus or perithymic fat surrounding the tumour (microscopically confirmed) –Macroscopic adherences to pleura or pericardium without invasion
III	–Microscopic invasion of the mediastinal pleura, visceral pleura or pericardium –Direct invasion into the lung parenchyma –Invasion into the phrenic or vague nerves –Invasion into or penetration through major vascular structures –Adherence (i.e. fibrous attachment) of lung or adjacent organs only if there is mediastinal pleural or pericardial invasion (microscopically confirmed)
IVA	Microscopically confirmed separate nodules in the visceral or parietal pleural, pericardial or epicardial surfaces
IVB	Lymphogenous or hematogenous metastasis

Scientific societies have recently proposed using the tumour–node–metastasis (TNM)-based staging system for TET, based on an analysis of OS using a retrospective international database of more than 10,000 cases [V, A] [16] (Table 4). The WHO classification correlates with both, the Masaoka-Koga and the 8th edition of the TNM staging classification, showing higher risk of advanced stages with B3 thymoma and TC [17].

**Table 4:** Tumour–node–metastasis staging [16]

T	T1	Tumour encapsulated extending into the mediastinal fat; may involve the mediastinal pleura
	T1a	Tumour with no mediastinal pleural involvement
	T1b	Tumour with direct invasion of mediastinal pleura
	T2	Tumour with direct invasion of pericardium
	T3	Tumour with direct invasion of lung, brachiocephalic vein, superior vena cava, chest wall, phrenic nerve, hilar (extrapericardial) pulmonary vessels
	T4	Tumour with invasion of aorta, arch vessels, intrapericardial artery, myocardium, trachea, oesophagus
N	N0	No regional lymph nodes
	N1	Metastasis in anterior (perithymic) nodes
	N2	Metastasis in deep intrathoracic or cervical lymph nodes
M	M0	No pleural, pericardial or distant metastases
	M1a	Separate pleural or pericardial nodule(s)
	M1b	Pulmonary intraparenchymal nodule or distant organ metastases

Stage	T	N	M
I	T1	N0	M0
II	T2	N0	M0
IIIA	T3	N0	M0
IIIB	T4	N0	M0
IVA	Any T	N1	M0
	Any T	N0,1	M1a
IVB	Any T	N2	M0, 1a
	Any T	Any N	M1b

Currently, the use of the TNM system as a guide to therapy has yet to be assessed, therefore, Masaoka-Koga staging is still the standard system in the routine management of patients, particularly for adjuvant radiotherapy, although both staging systems must be applied.

## Therapeutic strategies

It is strongly recommended that the treatment of patients with TET be discussed in multidisciplinary tumour boards (MTB). This is particularly important when assessing resectability, even in locally advanced disease, as feasibility is mostly based on the surgeon’s expertise [IV, B]. The MTB is even more important in rare cancers, as clinical expertise is more likely to be limited, and evidence-based decision-making is difficult [18–20].

### Surgery

The treatment of TET is based on complete resectability of the tumour. If it is achievable, upfront surgical resection is the cornerstone of treatment in T1–T3 (IIIA) according to the 8th edition of the TNM classification [IV,A] and certain patients with stage IVA (specially thymoma) [V,B] [21]. In thymoma, 10 year OS rates after surgery clinically meaningful—90% and 70% for stage I and II, and 55% and 35% for stage III and IVA, respectively. Patients who undergo complete resection achieve significantly better survival [22].

The standard approach is median sternotomy [IV, A]. Complete thymectomy (tumour, the residual thymus gland, and perithymic fat) is generally the preferred surgical approach to reduce risk of recurrence [23] [IV, B]. If the tumour is widely invasive (stage III and selected patients with IVa) en bloc removal of all affected structures should be performed [IV, A]. In this scenario, upfront induction chemotherapy should be discussed, especially in TC. Complete resection positively impacts outcomes [24, 25]; however, OS and disease free survival (DFS) are worse in TC. The areas with uncertain resection margins should be marked with clips to allow accurate delivery of postoperative radiotherapy [21, 25] [IV, B]. Phrenic nerve preservation does not impact survival, but increases the risk of local recurrence, so the benefit should be weighed up, particularly in patients with MG [26] [IV, C]. Minimally invasive surgery carried out by fully trained thoracic surgeons is only an option in stage I and possibly certain stage II tumours (< 5 cm and no sign of invasion of intrathoracic vessels, lung, pericardium or trachea) [IV, B] [27].

Although the impact of lymphadenectomy on survival in TET has not been demonstrated, following the new TNM classification routine lymphadenectomy of mediastinal and anterior cervical lymph nodes is recommended [16] [IV, A]. For stage III/IV tumours, sampling of other regions (paratracheal, aortopulmonary window and subcarinal area) is advised [IV, B]. Complete dissection (N1 + N2) is recommended in TC, due to the high rate of lymphatic spread (20% versus 3% in thymomas) [V, B] [28].

Finally, surgery also plays a role in the recurrences of TET [IV, A], as complete resection of recurrent lesions is associated with favourable outcomes [21].

### Radiotherapy

Postoperative radiotherapy (PORT) for TET has been associated with longer OS, particularly in stage IIB to III disease and positive margins (R1) [29]. However, although a beneficial effect of PORT in terms of recurrent free survival and OS has been shown in TC [21], the role of PORT is more controversial in T, even in stage III, and there is one ongoing prospective clinical trial testing PORT in this setting (RADIO-RYTHMIC trial, NCT04731610).

**Fig. 1:**
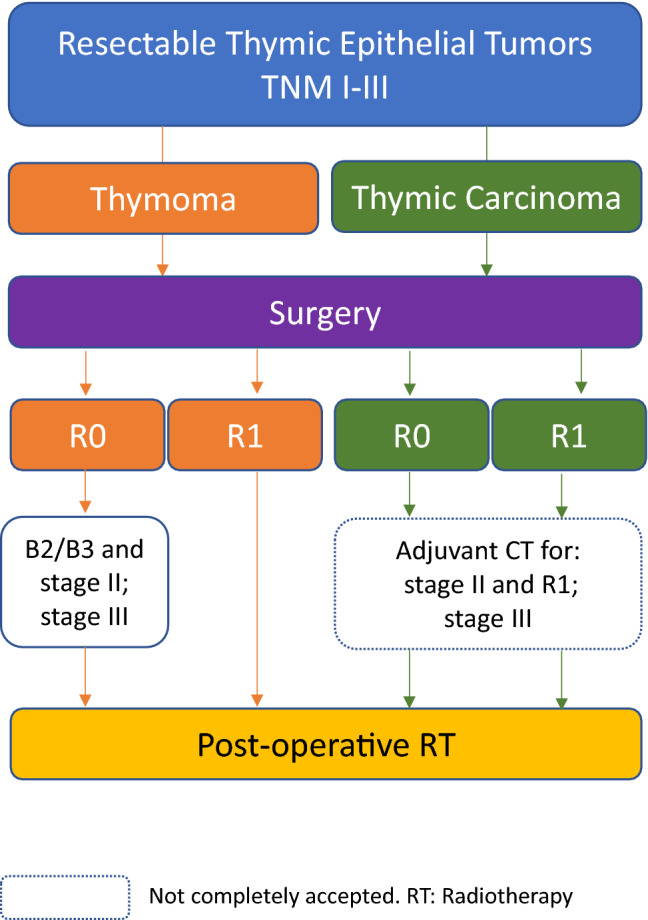
Management of patients with thymic epithelial tumours and resectable disease

Currently, PORT for T is indicated in stage III/IVA [IV, B], as well as in R1 or R2 resection irrespective of the stage. It can be proposed in stage IIB in patients with aggressive histology, such as B2/B3 thymoma [IV, C]. Regarding TC, postoperative RT is optional in stage I [V, C], and should be considered in all other stages [IV, B], as well as in patients undergoing R1 or R2 resection [IV, B] (Fig. 1).

Regarding postoperative RT in TET, it is recommended to use: (1) 3D conformal radiotherapy or intensity-modulated radiotherapy, which has become more widely available and better preserves normal lung and heart (III, A). (2) A total dose of 45–50 Gy after complete resection, 56 Gy after R1 resection, with a boost to areas of likely residual disease, marked with clips [IV, B]. The treatment volume may include the involved nodes [IV, B] and the site of a resected pleural implant [IV, C]. (3) Conventional fractionation scheme consisting of daily doses of 1.8–2 Gy over 4–6 week period. Postoperative radiotherapy should start within 3 months of the surgical procedure [IV, B].

**Fig. 2:**
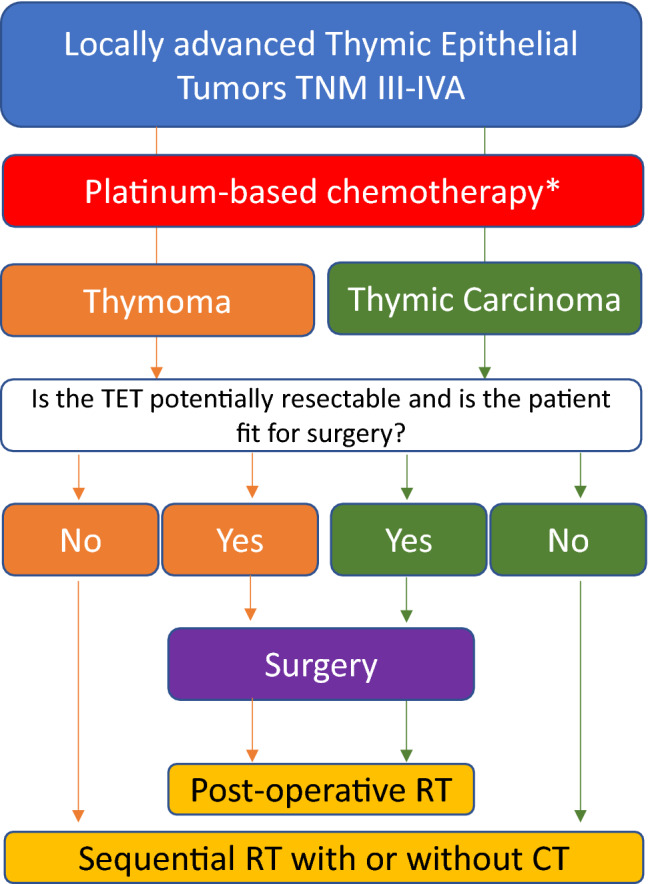
Management of patients with thymic epithelial tumours and locally advanced disease

At diagnosis, nearly 30% of patients with TET have unresectable locally advanced disease. In these cases, RT can be delivered concurrently with chemotherapy (platinum and etoposide) either as definitive treatment {dose of RT 60 to 66 Gy, [V, C]} or as a neoadjuvant approach followed by surgery [V, C]. In patients receiving induction chemotherapy, definitive sequential RT can be applied if the patient is not deemed a surgical candidate [III, A] [30] (Fig. 2). For those patients with induction chemotherapy followed by surgery, postoperative RT should be applied in case of TC, R1 or R2 resection [IV, B] and T with stage III [IV, B] or IIB if B2/B3 subtype [IV, C].

**Fig. 3:**
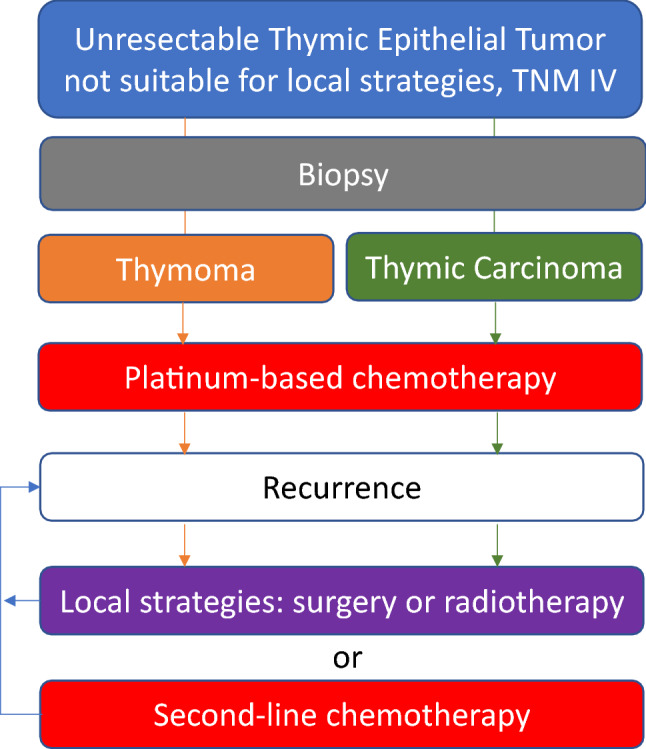
Management of patients with thymic epithelial tumours and advanced disease not suitable for local strategies

Finally, in unresectable local recurrences, exclusive radiotherapy may be useful (Fig. 3).

## Chemotherapy

### Perioperative setting

There is currently no indication for adjuvant chemotherapy in patients with R0 or R1 resection of thymoma [III, E]. Although still controversial, in TC adjuvant chemotherapy should be considered as an option for stage II with positive margins and stage III, in particular if chemotherapy has not used as induction treatment [V, C] [31] (Fig. 1).

Two to four cycles of induction chemotherapy is indicated in patients with stage III/IVA TET according to TNM classification [III, A] not suitable for upfront surgery (Fig. 2). Induction chemotherapy has shown a response rate (RR) of ~ 70%, and at least half of these patients may achieve a complete resection [19, 32]. Surgery should be offered to patients whom complete resection is deemed achievable after induction chemotherapy [III, A]. However, if R0 resection is not achievable or if the patient is not in good enough general condition for surgery, sequential definitive RT is recommended [III, A]. There is no standard chemotherapy [33], but the RR of cisplatin, doxorubicin and cyclophosphamide (PAC) is similar in T and in TC, and is associated with longer time to progression [19]. Other potential induction chemotherapy schedules include carboplatin and paclitaxel and cisplatin and etoposide [19, 33]. Although concurrent induction chemoradiotherapy may provide higher complete resection rates compared with induction chemotherapy alone, it is associated with a higher incidence of toxicity and postoperative complications [V, C] [32].

## Management of advanced disease

### First-line setting

In patients with metastatic disease with no intent of subsequent local treatment, definitive systemic chemotherapy is the standard of care [III, A]. Up to six cycles of cisplatin-based multi-agent combinations are recommended in this setting [21, 33], such as PAC (again, the most frequently used), carboplatin plus paclitaxel, and cisplatin plus etoposide, the former particularly in TC [19] (Table 5). Gemcitabine-platinum and paclitaxel platinum shows similar outcomes in TC [34]. In the metastatic setting, the RR with PAC was similar regardless of histologic subtype, but the RR with PAC were higher versus other regimens [19]. These strategies taken together provided a RR of ~ 30% and a median PFS of ~ 6 months [19] with prolonged median OS (more than 3 years) [33], suggesting that patients with TET may receive several subsequent treatment lines.

**Table 5:** Selected treatment regimens for advanced thymic epithelial tumours assessed in phase II trials

Regimen	Agents	Doses
PAC	Cisplatin	50 mg/m^2^ IV/Q3W
	Doxorubicin	50 mg/m^2^ IV/Q3W
	Cyclophosphamide	500 mg/m^2^ IV/Q3W
Carboplatin/paclitaxel	Carboplatin	AUC 5–6 IV/Q3W
	Paclitaxel	175–200 mg/m^2^ IV/Q3W
Cisplatin/etoposide	Cisplatin	60–75 mg/m^2^ d1/Q3W
	Etoposide	100 mg/m^2^ × 3 days IV/Q3W
VIP	Etoposide	75 mg/m^2^ × 4 days IV/Q3W
	Ifosfamide	1.2 g/m^2^ × 4 days IV/Q3W
	Cisplatin	20 mg/m^2^ × 4 days/Q3W
Pemetrexed	Pemetrexed	500 mg/m^2^ IV/Q3W
Capecitabine/gemcitabine	Capecitabine	650 mg/m^2^ bid × 14 days/Q3W
	Gemcitabine	1000 mg/m^2^ day 1 and 8 IV/Q3W
Oral etoposide	Etoposide	25 mg/8 h day 1–21/Q4W
Everolimus	Everolimus	5–10 mg/day, continuous
Sunitinib	Sunitinib	25–50 mg/day 1–28 Q6W
Lenvatinib	Lenvatinib	14–24 mg/day, continuous

*IV* intravenous, *Q3W* every 3 weeks, *Q4W* every 4 weeks, *Q6W* every 6 weeks

Recurrences in TET are not rare and should be managed as newly diagnosed tumours. In case of potentially resectable recurrent disease, surgery or other local strategies should be assessed instead of systemic treatment [35]. If not deemed possible, re-administration of a previously effective treatment must be discussed [IV, B], especially in case of previous response and late recurrence [36, 37]. Potential cardiac toxicity with anthracyclines and previous mediastinal radiotherapy should be taken into account in case of re-treatment with PAC (Fig. 3).

### Second-line and beyond

Owing to the rarity of this tumour, there is limited information about standard second-line chemotherapy, but subsequent treatment lines are indicated [III, B]. Although there is no significant difference in outcome between monotherapy and multidrug chemotherapy in TC [37], carboplatin and paclitaxel [III, B] is an accepted regimen in this setting regardless of the histologic subtype [19]. Other potential chemotherapeutic second-line schedules include pemetrexed [III, B], particularly in thymoma [38], oral etoposide [IV, B] [39] and gemcitabine and capecitabine [III, B] [40] (Table 5). The RR decreases with subsequent chemotherapy lines [19].

### Targeted therapies and immunotherapy

Although TET may have somatic mutations, a personalized treatment approach is challenging because these tumours are enriched by *HRAS, NRAS, TP53* and *GTF2I* mutations, with a limited number of actionable mutations suitable for a targeted therapy [41]. Several pathways involved in TET being explored as potential therapies in previously treated patients, such as tyrosine kinase inhibitors (TKI) and immune checkpoint inhibitors (ICI). However, none of these potential treatment strategies have received approval by the European Medcines Agency.

Tc-*KIT* mutation occurs in 10% of TC, however, the efficacy of c-*KIT* inhibitors is limited [5]. However, other multi-TKIs with antiangiogenic properties have reported clinical activity mainly in TC such as sunitinib at 50 mg, 4 weeks on 2 weeks off (RR: 26%, median PFS: 7.2 months) [III,A][5] and lenvatinib at 24 mg/day (RR: 39%, PFS 9.6 months) [42]. However, due to evidence of grade 3 cardiovascular toxicity leading to lenvatinib discontinuation in 17% of patients, it is recommended to initiate treatment with lenvatinib at 14 mg/day and prospectively increase the dose according to the tolerance [III, A]. Everolimus (10 mg/day), an mTOR TKI, has shown activity in TET with a disease control rate of 88% and median PFS of 10.1 months (16.6 months in T and 5.6 months in TC) [III, A] [5]. Careful toxicity monitoring is advised due to the potential risk of pulmonary toxicity with everolimus.

TET has the lowest mutational burden among adult cancers [41]; and only 6% metastatic TC have a burden > 10 mutations/megabase (mut/Mb), and 3% have > 20 mut/Mb [43]. In contrast, PD-L1 expression in TET ranges from 34 to 94% using different cut-off points [5]; however, the prognosis and predictive value of PD-L1 expression is unclear. This evidence prompted researchers to evaluate the role of ICI in TET, mainly in TC, as AIDs are uncommon in patients with TC. In two phase II studies, pembrolizumab showed a RR of ~ 20% and median PFS of ~ 5 months, and high PD-L1 expression correlated with better outcomes. However, immune-related adverse events (ir-AE) occurred in up to 20% of patients [5]. Although in a Japanese trial nivolumab did not show RR in TC [5], a recent phase II NIVOTHYM trial testing nivolumab (240 mg every 2 weeks) in B3 thymoma and TC (*N* = 55) reported a RR of 12%, with 52% of patients without progression at 6 months and a median OS of 21.3 months. The second cohort of the trial testing the combination of nivolumab plus ipilimumab is ongoing [44]. Finally, in a phase I trial, avelumab showed efficacy in 7 cases of T (RR of 29%), but was accompanied by an unacceptable high frequency of ir-AEs [5]. The efficacy of the combination of ICI and anti-angiogenics is being tested in several clinical trials (NCT04710628, NCT03463460). All these data suggest that ICI may play a role in TET, although it is not currently the standard of care. Indeed, there is strong evidence that patients with T or AIDs should not receive ICI, and off-label administration of ICI should only be performed under strict monitoring.

## Surveillance

While a relapse might still be suitable for radical treatment, patients benefit from regular radiological assessment. Potential recommendations are [V, C] [21]:

- (1) Baseline CT scan within 3–4 months after surgical resection.
- (2) For completely resected stage I–II thymoma: annual CT scan for 5 years, then every 2 years.
- (3) For stage III–IV thymoma, thymic carcinoma or after R1 or R2 resection: CT scan every 6 months for 3–5 years, thereafter annually.
- (4) Continue follow-up for 10–15 years.

A flare-up of AIDs may suggest tumour recurrence, and early radiological assessment to rule out recurrence is recommended.

Patients with TETs should be encouraged to get vaccinated against COVID-19 with mRNA vaccines. Tolerability in patients with TETs is comparable to the general population, and 15% of patients developed mild flare-up of AID after some of two injections [45].
